# Interhemispheric Interneuron Activity Changes Induced by Bilateral and Unilateral Finger Movements in Stroke Subjects

**DOI:** 10.14789/ejmj.JMJ25-0028-OA

**Published:** 2025-12-05

**Authors:** KAORU HONAGA, MICHIYUKI KAWAKAMI, AIKO ISHIKAWA, MORITOMO MAEDA, MAMI TANI, YUHEI MURAKAMI, REINA ISAYAMA, TOSHIYUKI FUJIWARA

**Affiliations:** 1 Department of Rehabilitation Medicine, Juntendo University Graduate School of Medicine, Tokyo, Japan; 1 Department of Rehabilitation Medicine, Juntendo University Graduate School of Medicine, Tokyo, Japan; 2 Department of Rehabilitation Medicine, Keio University School of Medicine, Tokyo, Japan; 2 Department of Rehabilitation Medicine, Keio University School of Medicine, Tokyo, Japan

**Keywords:** inter-hemispheric inhibition, cortical plasticity, cerebrovascular disease, transcranial magnetic stimulation, rehabilitation

## Abstract

**Objectives:**

To assess the effect of unilateral and bilateral hand movements on interhemispheric inhibition (IHI), which was assessed with transcranial magnetic stimulation and compared between healthy volunteers and patients with stroke.

**Methods:**

Eleven patients with chronic hemiparetic stroke performed paretic and bilateral hand tasks, and six healthy volunteers performed right-hand and bilateral hand tasks. IHI between the hemispheres was assessed using transcranial magnetic stimulation before and 0, 10, and 30 min after both tasks.

**Results:**

IHI from contralesional to ipsilesional motor cortex (M1) after the bilateral hand task was disinhibited more strongly and for a longer time than that after the paretic hand task in stroke patients. IHI from ipsilesional to contralesional M1 in stroke patients was significantly disinhibited with the bilateral hand task and inhibited with the paretic hand task 10 min after the tasks. Task and time had no significant effects on IHI in both tasks in healthy volunteers.

**Conclusions:**

Inter-hemispheric inhibitory interneurons were modified differently by unilateral and bilateral hand movements. Bilateral hand movement might have a stronger effect on IHI than paretic hand movement.

## Introduction

Interhemispheric interneurons, which connect homologous cortical areas of the two cerebral hemispheres, play crucial roles in the transfer of several types of information, such as on sensory, motor, and cognitive functions.

In healthy persons, these interhemispheric interneurons in the motor cortex are mediated via the largest fiber bundle tract in the brain, the corpus callosum^1, 2)^, and it suppresses the abnormal contralateral brain activities, such as mirror movement, during the production of voluntary unilateral movement. By this suppression, people can perform appropriate movements for their tasks in their daily activities^3, 4)^.

The effect of altered interhemispheric inhibition (IHI) between homologous motor cortical areas after stroke has been previously reported to contribute to residual hemiparesis^5)^. The inhibition from the affected hemisphere to the unaffected hemisphere decreases early after stroke, and this disinhibition changes over the time course^6, 7)^. This disinhibition of IHI is seen only in patients with lesions including interhemispheric interneurons.

Over the time course from stroke onset, it has been suggested that the inhibitory and facilitatory circuits of affected and unaffected hemispheres are changed, especially in subcortical stroke^8)^.

According to the hypothesis of interhemispheric imbalance in stroke, it is supposed that reduced corticospinal excitability in the affected hemisphere decreases IHI from the affected hemisphere to the unaffected hemisphere and increases the excitability of the unaffected hemisphere. Increased motor cortex excitability, then, increases IHI from the unaffected hemisphere to the affected hemisphere. The change of IHI, furthermore, decreases motor cortex excitability in the affected hemisphere and inhibits recovery from stroke. Based on this interhemispheric imbalance hypothesis, many studies applied noninvasive neuromodulation to the affected hemisphere to reduce the motor cortex excitability in the unaffected hemisphere. However, despite the large number of studies, different groups have reported highly variable and even opposite results^9-11)^. The concept of interhemispheric imbalance is likely to be too simplistic to be successfully applied to each patient^12)^.

A previous study reported that excessive unilateral exercise is effective for the motor recovery of the paretic hand^13)^, while bilateral hand exercise resulted in better improvement than unilateral movement^14)^. The effects of unilateral and bilateral upper extremity exercises on IHI in both healthy participants and patients with stroke have not been clarified. The objective of the present study was to assess the effects of unilateral and bilateral hand movements on IHI, which was assessed with transcranial magnetic stimulation^15)^ and compared between healthy participants and patients with strokes.

## Materials and Methods

Eleven patients (ten men and one woman) with chronic stroke were recruited for this study from the outpatient clinic. Their mean age was 67.2 ± 7.0 years, and their mean time from stroke onset was 57.0 ± 47.2 months (8-168 months, median 46 months). Inclusion criteria consisted of: 1) first-ever stroke; 2) age over 20 years; 3) time from onset more than six months; and 4) magnetic evoked potentials (MEPs) were elicited from the affected extensor digitorum communis (EDC) by stimulation of the affected hemisphere. Exclusion criteria were: 1) history of major psychiatric or previous neurological diseases, including seizure; 2) cognitive impairment precluding informed consent; and 3) use of drugs affecting the central nervous system.

Five healthy volunteers (3 men, 2 women) were also recruited for this study. Their mean age was 61.2 ± 3.8 years, and they were all right-handed. The exclusion criteria for healthy volunteers were the same as those for stroke patients. The purpose and procedures of the study were explained, and all participants provided their written informed consent. The study was approved by the institutional ethics review board and was registered to the University Hospital Medical Information Network (UMIN) clinical trial registry (UMIN000001956). The study was performed in accordance with the Declaration of Helsinki.

### Clinical evaluations

The paretic upper extremity was assessed using the Stroke Impairment Assessment Set (SIAS) motor test^16)^ and the upper extremity scores of the Fugl-Meyer assessment set^17)^. SIAS is a standardized measure of stroke impairment consisting of 22 subcategories. The paretic side motor functions of the upper extremity were tested with the knee-mouth test and the finger test. They were rated from 0 to 5, with 0 indicating complete paralysis and 5 no paresis. The score 1 for the finger test was divided into 3 subscales: 1a (mass flexion), 1b (mass extension), and 1c (minimal individual movement)

### Assessment of transcranial magnetic stimulation (TMS)

Participants were seated in a reclining chair with the elbow flexed at 70°. Surface electrodes were placed bilaterally on the skin areas overlying the EDC muscles in a bipolar montage (interelectrode distance, 2 cm). Before attaching the electrodes, the skin areas were rubbed with alcohol, and the skin resistance was kept below 5 kΩ. A Neuropack™ electromyography machine (Nihon Kohden Co. Tokyo, Japan) was used to record and analyze EMG data. The band pass filter was set at 30 Hz-2 kHz.

TMS was delivered with a Magstim 200 magnetic stimulator (The Magstim Company, Whitland, Dyfed, UK). Magnetic stimulation was applied over the primary motor cortex (M1) through a figure-of-eight coil having an external wing diameter of 9 cm and a peak magnetic field of 2.2 Tesla. TMS was delivered to the optimal scalp position for activation of the EDC muscle overlying the left and right M1. The stimulating coil was placed over the optimal site for eliciting responses in the EDC and oriented so that the current in the brain flowed in a posterior to anterior direction through this optimal stimulating site.

Before the examination, the resting motor threshold (RMT) was measured in all participants. The RMT was determined according to the recommendation of the International Federal of Clinical Neurophysiology (IFCN) Committee^18)^. It was defined as the intensity needed to evoke a minimal EMG response (>50 μV) in at least five of 10 trials in the relaxed EDC.

### Interhemispheric inhibition (IHI)

Participants were seated in a reclining chair with the elbow flexed at 70°.

Two figure-of-eight-shaped coils were connected directly to two Magstim 200 magnetic stimulators. Coils were positioned at an angle of 45° from the surface of the head.

Interhemispheric paired pulses were applied with interstimulus intervals (ISIs) of 10 msec and 15 msec. Conditioning and test stimulus intensity was 120% RMT of the respective side. At different intervals after the conditioning stimulus, a second stimulus was applied to the opposite M1 as a test stimulus.

Five trials were recorded for each ISI and single-pulse stimulation in pseudorandom order controlled by a laboratory computer. Stimuli were applied every 5 seconds. The conditioned MEP amplitudes were expressed as the ratio to the mean MEP amplitude with test stimulation given alone (IHI ratio). The IHI ratio was examined in both hemispheres. To assess the IHI from the contralesional M1 to the ipsilesional M1, a conditioning stimulus was applied to the contralesional M1, and a test stimulus was applied to the ipsilesional M1.

### Task procedure

Participants were asked to extend their fingers for five seconds and then relax for five seconds and repeat this for a total of 10 minutes, only with the paretic hand (paretic hand task) or bilateral fingers simultaneously (bilateral hand task). For the healthy volunteers, the unilateral hand task was performed by the right hand (right hand task).

**Figure 1:**
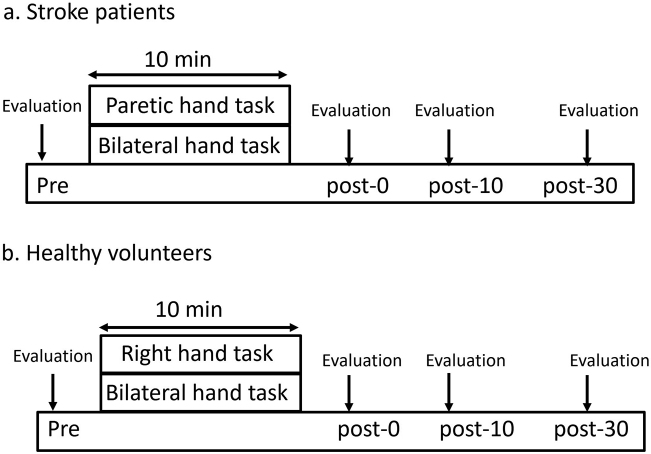
Experimental procedure: a. Stroke patients participated in the following two sessions: (1) Paretic hand task; (2) Bilateral hand task. IHI was measured at baseline (pre), immediately after (post-0), 10 min after (post-10), and 30 min after (post-30) each task. b. Healthy volunteers participated in the following sessions: (1) Right hand task; (2) Bilateral hand task.

IHI was assessed with TMS at baseline (pre) and immediately after (post-0), 10 min after (post-10), and 30 min (post-30) after the end of the task. All participants were assigned randomly to the two tasks at least three days after the preceding study (Figure 1).

### Data analysis and statistics

Two-factor repeated measures analysis of variance (ANOVA) was used to investigate the effects of task (paretic hand task and bilateral hand task for stroke patients, and right hand task and bilateral hand task for healthy volunteers) and time (pre, post-0, post-10, post-30) on IHI. Post hoc analysis was performed using the Bonferroni test to detect significant differences within and between tasks at each time point.

The Wilcoxon signed-rank test was used to compare differences in the RMT between before and after the task. All statistical analyses were performed with IBM SPSS Statistics 29.0.0.0 for Windows.

## Results

The participants’ characteristics are summarized in Table 1. All the participants (stroke group and healthy group) could perform the two tasks and the TMS study. No side effects were observed in these sessions.

**Table 1:** Patients’ data and clinical findings

Patient No.	Age (y)	Gender	Lesion	Infarct	Side	From on-set(M)	SIAS-pro	SIAS-dis	F-M
1	62	Male	Isch	Cort-sub	Right	168	4	4	29
2	65	Male	Hem	Subcort	Right	66	3	1b	25
3	60	Male	Isch	Subcort	Right	60	3	1b	31
4	71	Male	Isch	Subcort	Right	8	2	1b	25
5	55	Male	Hem	Subcort	Right	50	4	4	56
6	72	Male	Isch	Subcort	Right	46	4	2	37
7	75	Male	Isch	Cort-sub	Right	115	3	1c	41
8	72	Female	Isch	Subcort	Left	15	2	1c	28
9	78	Male	Isch	Subcort	Right	16	2	1c	51
10	65	Male	Isch	Subcort	Left	41	3	3	54
11	64	Male	Isch	Subcort	Right	42	3	1b	17

Abbreviations: Cort, cortical; Subcort, subcortical; Isch, ischemic; Hem, hemorrhagic; SIAS-pro, SIAS Knee-Mouth Test; SIAS-dis, SIAS Finger-Function Test; F-M, upper score of Fugl-Meyer assessment set

### Reproducibility of the resting motor threshold

Regarding the reproducibility of the RMT before the intervention between the two days, the intraclass correlation coefficient was 0.92 in the affected hemisphere and 0.85 in the unaffected hemisphere. Table 2 shows the RMT change before and after the two tasks. There were no significant differences between before and just after the intervention in both unilateral and bilateral hand movements (Table 2).

**Table 2:** Changes of the resting motor threshold between pre task and post task

	Unilateral movement				Bilateral movement		
	pre	post	p*		pre	post	p*
Stroke group							
affected hemisphere	63.2 ± 6.5	62.5 ± 6.7	n.s		63.0 ± 8.0	63.7 ± 7.1	n.s
unaffected hemisphere	48.2 ± 8.0	49.3 ± 8.0	n.s		47.7 ± 8.6	48.1 ± 8.5	n.s
Control group							
Left hemisphere	55.0 ± 6.1	55.2 ± 6.1	n.s		53.8 ± 7.1	53.4 ± 6.8	n.s
Right hemisphere	54.0 ± 8.1	54.4 ± 7.4	n.s		54.8 ± 7.8	54.0 ± 6.6	n.s

* Wilcoxon signed rank test p < 0.05

### IHI in the stroke group

#### IHI from the contralesional M1 to the ipsilesional M1 (Figure 2a)

In the stroke group, the values of IHI from the contralesional M1 to the ipsilesional M1 with the paretic hand task were 0.89 (0.12) at pre, 0.92 (0.13) at post-0, 1.05 (0.14) at post-10, and 0.87 (0.10) at post-30. The values of IHI from the contralesional M1 to the ipsilesional M1 with the bilateral hand task were 0.88 (0.09) at pre, 1.09 (0.16) at post-0, 1.05 (0.14) at post-10, and 1.06 (0.17) at post-30.

Two-factor repeated measures ANOVA showed a significant interaction between task (paretic hand task and bilateral hand task) and time (pre, post-0, post-10, post-30) on IHI from the contralesional to the ipsilesional hemisphere (F_(__3,80__)_ = 3.5293; p = 0.019). The post hoc Bonferroni test showed that IHI at post-10 differed significantly from post-30 after the paretic hand task, and IHI at baseline differed significantly from those at post-0, post-10, and post-30 after the bilateral hand task. Comparing the paretic hand task and the bilateral hand task, changes in IHI were significant at post-0 (p = 0.013) and post-30 after (p = 0.006) the bilateral hand task.

**Figure 2:**
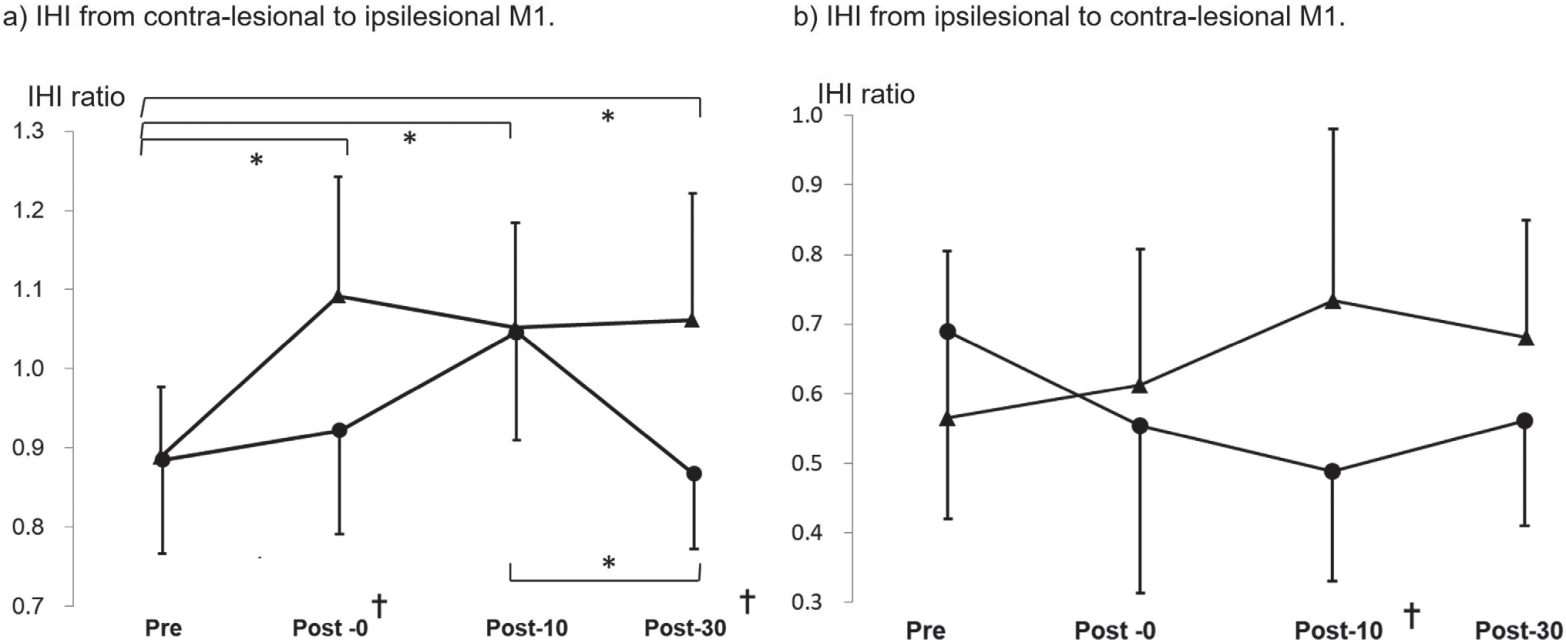
IHI in stroke patients (a) IHI from the contralesional to the ipsilesional hemisphere, circle: unilateral hand task, triangle: bilateral hand task. (b) IHI from the ipsilesional M1 to the contralesional M1, circle: unilateral hand task, triangle: bilateral hand task. * post hoc Bonferroni test of the time factor (p < 0.05). †post hoc Bonferroni test of the task factor (unilateral and bilateral hand tasks) (p < 0.05).

#### IHI from the ipsilesional M1 to the contralesional M1 (Figure 2b)

In the stroke group, the values of IHI from the ipsilesional M1 to the contralesional M1 with the paretic hand task were 0.67 (0.27) at pre, 0.56 (0.24) at post-0, 0.46 (0.16) at post-10, and 0.56 (0.15) at post-30. The values of IHI in the unaffected hemisphere with the bilateral hand task were 0.56 (0.22) at pre, 0.61 (0.18) at post-0, 0.72 (0.23) at post-10, and 0.67 (0.16) at post-30.

Repeated measures ANOVA showed a significant interaction between task (paretic hand task and bilateral hand task) and time (pre, post-0, post-10, post-30) in IHI from the ipsilesional M1 to the contralesional M1 (F_(__3,80__)_ = 2.877; p = 0.041). The post-hoc Bonferroni test showed no significant differences in IHI between baseline and other time points after the paretic and bilateral hand tasks. Comparing the paretic and bilateral hand tasks, changes in IHI were significant at post-10 after the bilateral hand task (p = 0.008).

### IHI in healthy volunteers

**Figure 3:**
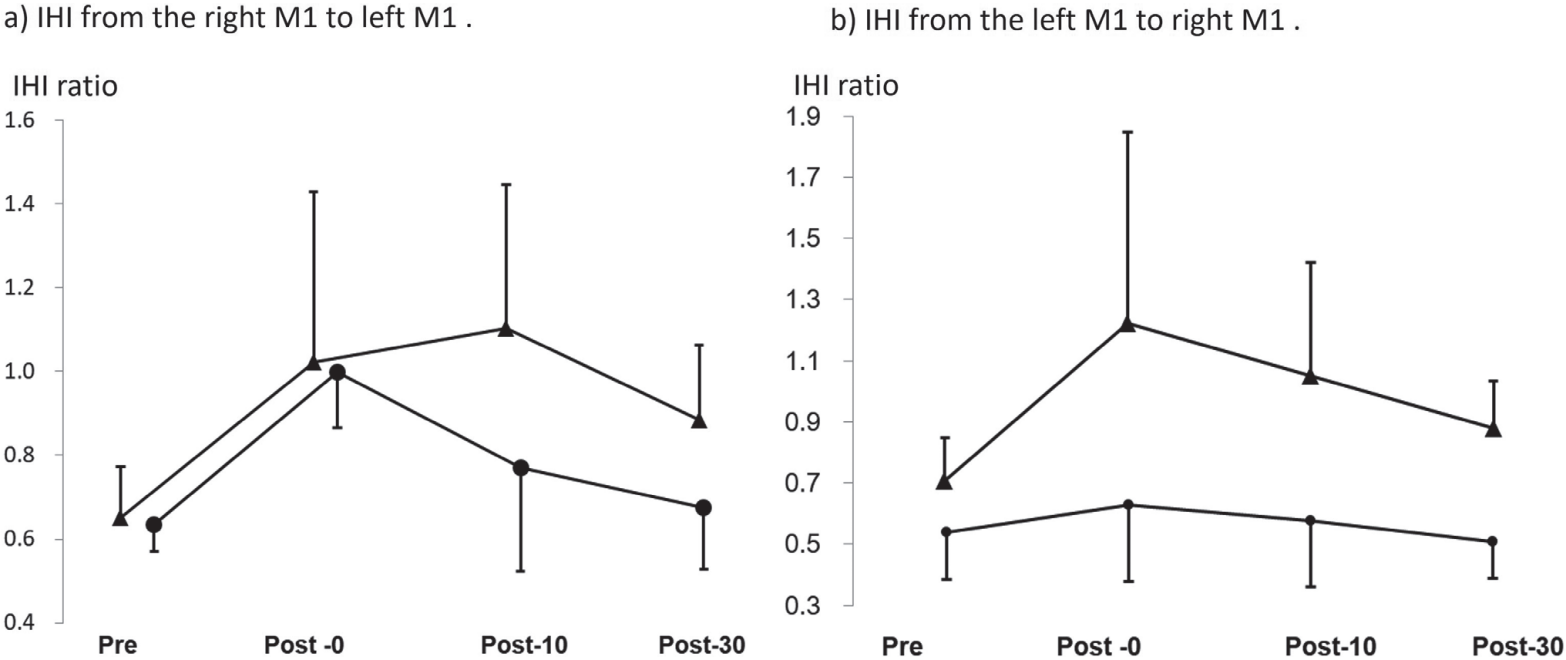
IHI in healthy volunteers (a) IHI from the right M1 to the left M1, circle: right hand task, triangle: bilateral hand task. (b) IHI from the left M1 to the right M1, circle: right hand task, triangle: bilateral hand task.

In the healthy volunteers, mean values of IHI from the right M1 to the left M1 were 0.63 (0.07) at pre, 0.99 (0.13) at post-0, 0.77 (0.25) at post-10, and 0.67 (0.15) at post-30 with the right hand task, and then 0.64 (0.12) at pre, 1.01 (0.40) at post-0, 1.09 (0.34) at post-10, and 0.87 (0.18) at post-30 with the bilateral hand task (Figure 3a). IHI from the right M1 to the left M1 seemed to decrease after both tasks. However, two-factor repeated measures ANOVA did not show a significant effect of task (paretic hand task and bilateral hand task) and time (pre, post-0, post-10, post-30) (F_(__3,24__)_ = 0.873; p = .469).

The mean values of IHI from the left M1 to the right M1 were 0.67 (0.17) at pre, 0.77 (0.27) at post-0, 0.71 (0.23) at post-10, and 0.64 (0.13) at post-30 with the right hand task, and then 0.71 (0.14) at pre, 1.22 (0.63) at post-0, 1.05 (0.37) at post-10, and 0.88 (0.15) at post-30 with the bilateral hand task in healthy volunteers.

Two-factor repeated measures ANOVA did not show a significant effect of task (paretic hand task and bilateral hand task) and time (pre, post-0, post- 10, post-30), (F_(3,24)_ = 0.690; p = .567) (Figure 3b).

## Discussion

This study assessed the effects of paretic and bilateral hand movements on IHI of chronic stroke patients and showed the changes in IHI and their effects on the duration of disinhibition of IHI.

Paretic finger extension decreased IHI from the contralesional hemisphere to the ipsilesional hemisphere and increased motor cortex excitability of the ipsilesional hemisphere, and bilateral finger movement decreased IHI in both directions in stroke patients, whereas the change of IHI was not significant in healthy volunteers.

### Bilateral and unilateral hand movements

The present results suggest that bilateral finger movement increased motor cortex excitability more than unilateral finger movement in stroke patients. The disinhibition of IHI from the contralesional hemisphere to the ipsilesional hemisphere in the stroke group was prolonged after the bilateral hand movement. The IHI from the contralesional hemisphere to the ipsilesional hemisphere was significantly disinhibited 30 min after bilateral hand movement in the stroke group. The IHI from the ipsilesional hemisphere to the contralesional hemisphere in the stroke group and the IHI from the right hemisphere to the left hemisphere in the healthy group were significantly changed, but the lasting effect of this IHI was weaker than that of the IHI from the contralesional hemisphere to the ipsilesional hemisphere. A previous study reported that, in stroke patients, increasing MEP in the paretic hand after bimanual pinch effort was significantly greater than in healthy volunteers^19)^. In stroke patients, cortical plasticity differs from that of healthy persons, and the change of cortical activity during and after bilateral exercise might be stronger than that of healthy people. In the present study, bilateral hand movement in stroke patients enhanced cortical excitability and disinhibited bilateral IHI more than right hand movement and bilateral hand movement in the healthy group. These results indicate that cortical activity in stroke patients may be more sensitive to movement than in healthy people.

There are some reasons why bilateral hand movement changed ipsilesional cortical activities more than paretic hand movement. One of the reasons is the cross-talk effect of interhemispheric connections via the corpus callosum^20)^. Each hemisphere interferes with the other hemisphere and each usually inhibits the activities of the other one during unilateral movement. However, it is suggested that there are some neurons that specifically respond to simultaneous bilateral movements^21-23)^. Then, during bilateral hand movement, reinforcement occurs through neural crosstalk by these specific neurons, and this crosstalk might decrease the IHI from the contralesional hemisphere to the ipsilesional hemisphere.

The other reason is the effect of the homologous fibers to the muscle. Humans have a natural tendency towards symmetrical contraction of homologous muscles, which are known to require less cortical activation than unilateral movements^24, 25)^. It has been suggested that strictly unilateral motor movement requires complex interhemispheric interactions between a wide range of cortical areas. These interactions are needed to restrict motor output to the contralateral primary motor cortex that controls the intended hand movement^3)^.

Murase el al. reported that the ipsilateral M1 facilitates the contralateral M1 just before the onset of unilateral movement in healthy adults, but in stroke patients, just before the paretic hand movement, the unaffected M1 inhibits the affected M1^5)^. Steiners et al. also showed that, in a functional MRI study, bilateral movement enhanced activation in the primary motor cortex (M1) of the affected hemisphere compared with unilateral paretic hand movement in acute stroke patients. Then, after recovery, activation of M1 in the affected hemisphere did not differ between unilateral paretic and bilateral hand movements^26)^. In this study, the participants were patients in the acute phase of stroke with hemiparesis not so severe. Therefore, among patients with severe hemiparesis, there is a possibility that bilateral movements enhanced brain activities more in the affected hemisphere than unilateral paretic hand movement even in the chronic phase of stroke.

### Clinical application

There are many studies of neuromodulation techniques in current stroke rehabilitation, including noninvasive brain stimulation. Furthermore, it has been suggested that intensive physical therapy combined with other neuromodulation techniques, such as transcranial direct current stimulation, TMS, botulinum toxin injection, and robot therapy is effective for chronic stroke patients^27, 28)^. Furthermore, there are a few studies about how stroke patients practice with their paretic and non-paretic hands^26, 29-34)^.

However, the effectiveness of bilateral upper extremity movements in clinical rehabilitation is not clear. Some reports suggested that bilateral upper extremity movement is more effective than unilateral upper extremity exercise^29)^, some reports suggested that bilateral upper extremity movements are less effective^26, 32)^, and others suggested that bilateral and unilateral upper extremity exercises are similarly effective^30)^. These results might depend on the severity of the upper limb paresis, the time of intervention post-stroke, and the task^30)^. It might be that the required cortical excitability varies with the type of exercise, since some tasks may require high cortical activities and others do not. Thus, we have to select the appropriate exercise for rehabilitation. At the present time, the concept of the relationship between hand movement and cortical activity could help choose the appropriate exercise. In the current trends in neuro- rehabilitation, our findings might play an important role.

### Limitations and problems

There are some limitations and problems that remain.

Stroke patients in whom MEPs could be elicited by stimulation of the affected M1 by TMS were recruited. The reason for this is that the affected IHI was evaluated in this study. If the lesion were large and severe, the affected MEPs would be difficult to elicit, so that our participants might have the capacity for neural plasticity. Therefore, it is difficult to apply our results to all stroke patients. Furthermore, it is difficult to explain many complex hand movements from only the perspective of IHI. The affected IHI was disinhibited and prolonged by bilateral hand movement, but it is not clear whether this change is beneficial for motor recovery or not. In stroke rehabilitation, inappropriate exercise causes maladaptive motor plasticity^35)^. Regarding intracortical inhibition, abnormal disinhibition in the unaffected hemisphere persisted in patients whose motor function remained poor^36)^. Bilateral movements disinhibited not only the affected IHI, but also the unaffected IHI, so further investigation is necessary to judge whether the change is beneficial. In the future, it will be necessary to investigate the relationship between this change of the affected IHI and the motor recovery that results from rehabilitation.

In conclusion, the present study provided further evidence of the effect of unilateral and bilateral hand movements on IHI in chronic stroke patients. The IHI from the contralesional hemisphere to the ipsilesional hemisphere was disinhibited after both unilateral and bilateral hand movements. Comparing both tasks, bilateral hand movement strongly disinhibited the IHI from the contralesional hemisphere to the ipsilesional hemisphere, and the duration of the effect was longer. It is necessary to examine how this change is involved in the improvement of motor function in the future.

## Author contributions

KH and TF led the study, designed the study, performed data measurement and analysis, and wrote the manuscript. KH and MK performed data measurement and analysis. AI, MM, MT, YM and RI provided advice about data analysis and the manuscript.

## Conflicts of interest statement

The authors declare that there are no conflicts of interest.
